# Evaluation of Operated Dextro-Transposition of Great Arteries Patients in Follow-Up: Comparison of Transthoracic Echocardiography and Cardiac CT Angiography

**DOI:** 10.3390/diagnostics15192419

**Published:** 2025-09-23

**Authors:** Ali Nazım Güzelbağ, İsa Özyılmaz, Demet Kangel, Osman Nuri Bayrak, Hatice Dilek Özcanoğlu, Behzat Tüzün, Ali Can Hatemi, Erkut Öztürk, Serap Baş

**Affiliations:** 1 Department of Pediatric Cardiology, Saglik Bilimleri University, Basaksehir Cam and Sakura City Hospital, 34480 Istanbul, Turkey; isaozyilmaz@gmail.com (İ.Ö.); demetkangel@gmail.com (D.K.); erkut_ozturk@yahoo.com (E.Ö.); 2 Department of Radiology, Saglik Bilimleri University, Basaksehir Cam and Sakura City Hospital, 34480 Istanbul, Turkey; bayrak.5343@gmail.com (O.N.B.); serapbas579@gmail.com (S.B.); 3 Department of Anesthesiology, Saglik Bilimleri University, Basaksehir Cam and Sakura City Hospital, 34480 Istanbul, Turkey; dilekmersin@hotmail.com; 4 Department of Pediatric Cardiovascular Surgery, Saglik Bilimleri University Basaksehir, Basaksehir Cam and Sakura City Hospital, 34480 Istanbul, Turkey; drbehzattuzun@gmail.com (B.T.); alicanhatemi@gmail.com (A.C.H.)

**Keywords:** transposition of great arteries, arterial switch operation, computed tomography, echocardiography, congenital heart disease

## Abstract

**Background:** Arterial switch operation (ASO) is the standard surgical treatment for dextro-transposition of great arteries (d-TGA). Long-term complications affecting pulmonary arteries, coronary arteries, and aortic root necessitate detailed surveillance, but the optimal imaging strategy remains undefined. **Methods:** We retrospectively analyzed 47 patients with d-TGA who underwent ASO between January 2023 and June 2025 with at least six months postoperative follow-up. All patients underwent both transthoracic echocardiography (TTE) and ECG-gated cardiac CT angiography (CTA). Anatomical measurements, functional parameters, and diagnostic completeness were compared between modalities. **Results:** Median age at follow-up was 37.2 months. CT detected pulmonary artery stenosis in 31 patients (65.9%) and aortic root dilatation in 31 patients (65.9%). TTE provided incomplete pulmonary artery assessment in 11 patients (23.4%) and incomplete coronary evaluation in 13 patients (27.6%), while CT successfully evaluated all patients (100%). Strong correlation was found between left pulmonary artery bending angle and aortic root dimensions (r = 0.65, *p* = 0.016), suggesting mechanical interdependence of post-surgical anatomical changes. Median radiation exposure was 2.684 mSv (IQR: 1.5–4.6). During follow-up, 10 patients (21.3%) required cardiovascular interventions, with CT providing complete pre-intervention assessment in all cases. **Conclusions:** TTE alone is insufficient for complete anatomical assessment following ASO. An integrated imaging approach utilizing TTE for functional assessment and CT for anatomical evaluation optimizes post-ASO surveillance.

## 1. Introduction

Dextro-transposition of the great arteries (d-TGA) represents the most frequent cyanotic congenital heart disease in neonates, occurring in approximately 20–30.5 per 100,000 live births with a strong male predominance [1]. It accounts for 5–7% of all complex congenital heart defects and requires urgent neonatal intervention, as mortality approaches 30% within the first week of life and 90% within the first year without surgical correction [2,3].The arterial switch operation (ASO), first successfully performed by Adib Jatene in 1975, has become the preferred surgical treatment for d-TGA since the 1980s [4]. When combined with the Lecompte maneuver (described in 1981), ASO preserves the morphological left ventricle as the systemic ventricle. This approach avoids the multiple atrial incisions required in earlier atrial switch procedures [5,6]. Contemporary perioperative mortality rates have decreased to less than 5%, with excellent long-term survival exceeding 90% [7,8].

Despite these outstanding results, the growing population of ASO survivors reaching adulthood faces ongoing late complications requiring lifelong surveillance [9]. Recent large cohort studies demonstrate significant long-term complications by age 35 years. Cumulative reintervention rates reach 36% for the right ventricular outflow tract and pulmonary branches, while left ventricular outflow tract interventions occur in 16% of patients. Additionally, 11% require electrophysiological interventions, and 8% experience major clinical events including heart failure, endocarditis, and myocardial infarction [10].

The most frequent late complications include pulmonary artery stenosis (affecting 50–80% of patients) neo-aortic root dilatation (present in the majority of adults), and coronary artery complications (occurring in 3–11% of cases). These complications may necessitate reintervention via transcatheter or surgical approaches, making detailed imaging surveillance critical for optimal long-term outcomes [11,12,13,14,15,16].

Transthoracic echocardiography (TTE) remains the primary imaging modality for postoperative surveillance. It offers widespread availability, portability, safety, and real-time functional assessment. However, TTE has recognized limitations in providing complete anatomical evaluation after ASO, particularly for pulmonary arteries and coronary anatomy, where acoustic window limitations and complex post-surgical geometry may compromise diagnostic accuracy [17,18].

Recent advances in cardiac computed tomography have enabled high-resolution anatomical assessment. ECG-gated techniques with dose optimization protocols substantially reduce radiation exposure. Current guidelines from both the American College of Cardiology/American Heart Association and the European Society of Cardiology recommend consideration of coronary CT angiography for comprehensive post-ASO surveillance, though the optimal imaging strategy remains to be defined [19,20,21,22].

This study aimed to systematically evaluate the comparative diagnostic capabilities of TTE and cardiac CT angiography in postoperative d-TGA follow-up. We focused on defining their complementary roles in anatomical and functional assessment for comprehensive surveillance. We hypothesized that CT would demonstrate superior anatomical assessment capabilities, particularly for pulmonary arteries and coronary anatomy. While TTE remains valuable for functional evaluation, an integrated imaging approach would optimize post-ASO care.

## 2. Materials and Methods

Our study was a single-center, retrospective study. The study was designed in accordance with the tenets of the Declaration of Helsinki and obtained the necessary approval from the local ethics committee (Protocol Code: 2025/251). The study included patients diagnosed with d-TGA who underwent ASO between January 2023 and June 2025 with at least six months of postoperative follow-up. All cardiac CT angiography examinations were performed as part of routine clinical surveillance protocol at our institution for post-ASO patients, not for research purposes. Our standard practice includes systematic CT evaluation during early postoperative years to comprehensively assess complex post-surgical anatomy, particularly structures that may be inadequately visualized by echocardiography alone. Patients were systematically screened for eligibility based on predefined inclusion and exclusion criteria. Inclusion criteria required both TTE and ECG-gated cardiac CT angiography to be performed during the follow-up period. Exclusion criteria included postoperative follow-up periods of less than six months, complex additional cardiac anomalies significantly altering surgical approach, and missing CT or echocardiographic data. Of 52 consecutive patients who underwent ASO during the study period, 47 patients (90.4%) met inclusion criteria and comprised the final study cohort, with 5 patients excluded due to insufficient follow-up duration (*n* = 3), complex additional anomalies (*n* = 1), or incomplete imaging data (*n* = 1). The study protocol was approved by the institutional review board, and informed consent was obtained from parents or legal guardians.

Detailed demographic variables were documented, including age, sex, height, weight, and body surface area calculated using the Du Bois formula. We recorded gestational age, prenatal diagnosis, and preoperative balloon atrial septostomy. Surgical variables included age at ASO and birth weight. Current anthropometric measurements and Z-scores were calculated using normative data adjusted for age and sex.

All cardiac CT angiography examinations were conducted utilizing a 640-detector single-source computed tomography system (Aquilion ONE, GENESIS Edition; Canon Medical Systems, Otawara, Tochigi, Japan) equipped with a 16 cm wide-area detector array and integrated Adaptive Iterative Dose Reduction 3D Enhanced (AIDR 3D Enhanced) reconstruction algorithm. All imaging acquisitions employed prospective electrocardiographic gating within a single heartbeat. Data acquisition was performed using Volume Axial scanning mode with standardized parameters including 0.35 s gantry rotation time and scan coverage ranging from 80 to 160 mm, while tube current optimization was achieved through automated exposure control mechanisms [23]. Voltage selection followed a weight-based protocol to maximize iodine enhancement: 80 kV for patients weighing less than 35 kg and 100 kV for heavier patients. Contrast administration consisted of intravenous iodinated contrast material (Kopaq 350 mgI/mL; Onko&Kocsel Pharmaceuticals, Kocaeli, Turkey) delivered at 1.5 mL/kg body weight, immediately followed by a saline chaser bolus of 10–20 mL administered through a dual-syringe power injection system (MEDRAD, Bayer HealthCare, Beek, The Netherlands). Flow rates were individually optimized between 0.7 and 3.5 mL/s based on vascular access characteristics and patient anthropometrics. Full-strength contrast concentration was maintained throughout all injections. All examinations were conducted in free-breathing conditions without sedation requirements, under direct supervision of an experienced pediatric cardiac imaging specialist. For pediatric patients requiring sedation, intravenous midazolam (0.05–0.1 mg/kg) and/or fentanyl (1–2 mcg/kg) were administered by a cardiac anesthetist under continuous monitoring. Patients were monitored throughout the procedure with pulse oximetry, electrocardiography, and blood pressure monitoring. Recovery was supervised until patients returned to baseline neurological status. The timing strategy focused on capturing the first-pass contrast enhancement through cardiac and vascular structures, with the data acquisition window positioned at 45% of the cardiac cycle duration for patients demonstrating heart rates above 90 beats per minute [24]. Image acquisition was suspended during cardiac phases considered suboptimal for diagnostic quality. Following data collection, the attending radiologist retrospectively identified the cardiac phase demonstrating minimal motion artifacts while maintaining proximity to the predetermined optimal timing. Final image reconstruction utilized 0.5 mm slice intervals with standard reconstruction parameters and AIDR 3D Enhanced algorithm processing. All CT studies were analyzed by experienced cardiac radiologists, blinded to echocardiographic results.

Advanced image processing techniques were applied including multiplanar reformatting (MPR), maximum intensity projections (MIP), and three-dimensional volume rendering (VR) reconstructions. Comprehensive radiation exposure monitoring was performed for each patient, with systematic documentation of dose-length product (DLP), volumetric computed tomography dose index (CTDIvol), and anatomical coverage parameters. Patient radiation burden assessment incorporated both CT dose index measurements and dose-length product calculations. Effective dose estimations were computed using thoracic conversion coefficients according to the established formula: ED (mSv) = DLP (mGy.cm) × conversion factor (mSv.mGy^−1^.cm^−1^). Dosimetric calculations utilized CTDI and DLP measurements based on a 32 cm calibration phantom, with effective dose determinations adjusted for pediatric populations by applying a doubling factor to accommodate the 16 cm phantom reference standard. Effective doses were derived from chest conversion factors using the following formula: ED (mSv) = DLP (mGy.cm) × conversion factor (mSv.mGy^−1^.cm^−1^) [25]. Effective dose calculations were performed using age-specific conversion factors applied directly to DLP values. Age-specific conversion coefficients were applied for accurate dose estimation: 0.039 mSv/(mGy·cm) for neonates and infants, 0.026 mSv/(mGy·cm) for children 1–5 years, 0.018 mSv/(mGy·cm) for children 5–10 years, and 0.014 mSv/(mGy·cm) for children >10 years [24,26].

CT image analysis involved comprehensive evaluation of pulmonary arteries using multiplanar reformatted images. Systematic measurements of diameters were performed at standardized locations including main pulmonary artery at its widest point proximal to bifurcation, and branch PA diameters at three specific locations: proximal diameter immediately after bifurcation, pre-branching diameter before the first major segmental branch, and narrowest diameter at the point of maximum stenosis if present. Cross-sectional areas of all PA segments were calculated and indexed to body surface area. Novel geometric parameters were systematically measured including main PA axial angle calculated as the angle between neo-pulmonary and neo-aortic roots from the median plane, and PA bending angles measured as the outer angle between main PA axis and branch PA axes using multiplanar reconstruction. Pulmonary artery stenosis was defined using both morphological and quantitative criteria. Significant stenosis was considered present when the narrowest diameter Z-score was ≤−2.0 or when there was ≥50% reduction in cross-sectional area compared to the proximal reference segment. For branch pulmonary arteries, stenosis severity was graded as follows: mild stenosis (Z-score −2.0 to −2.5 or 25–49% area reduction), moderate stenosis (Z-score −2.5 to −3.0 or 50–74% area reduction), and severe stenosis (Z-score < −3.0 or ≥75% area reduction). Main pulmonary artery stenosis was defined as a Z-score ≤ −2.0 at the narrowest point proximal to bifurcation. Aortic stenosis was classified as supravalvular when sinotubular junction Z-score was ≤−2.0 with associated pressure gradient evidence from echocardiography, while aortic coarctation was defined as discrete narrowing with ≥50% diameter reduction compared to adjacent normal segments.

The thoracic aorta was thoroughly evaluated using multiplanar reconstructions with measurements at standardized anatomical landmarks including aortic annulus diameter during systole, sinus of Valsalva diameter at maximal dimension, sinotubular junction diameter at the narrowest point, ascending aorta diameter at the level of PA bifurcation, and aortic arch measurements at three levels with additional assessment for morphological abnormalities.

Detailed coronary artery assessment was performed using volume-rendered reconstructions and curved multiplanar reformation. Anatomical evaluation focused on systematic documentation of coronary ostial positions, branching patterns, and course abnormalities classified according to the Leiden classification system. The assessment included detailed evaluation of coronary ostial morphology, spatial relationships between reimplanted coronary arteries and surrounding structures, and evaluation for complications including stenosis, acute angulation, kinking, and inter-arterial course. Major aortopulmonary collateral arteries were systematically identified when present.

Echocardiographic assessment was performed using the Philips Affiniti 50 ultrasound system (Bothell, WA, USA) with high-frequency 9 and 12 MHz transducers optimized for pediatric imaging. Standardized pediatric echocardiographic views were acquired including parasternal long and short axis, apical four- and five-chamber, subcostal, and suprasternal views according to American Society of Echocardiography guidelines for congenital heart disease. Cardiac morphology was thoroughly assessed using a standardized segmental approach evaluating atrial situs, venoatrial connections, atrioventricular connections, ventricular anatomy, ventriculoarterial relationships, great artery spatial relationships, intracardiac defects, and extracardiac vascular anomalies. All echocardiographic studies were independently interpreted by experienced pediatric cardiologists with more than 10 years of specialized experience, blinded to CT findings.

Left ventricular systolic function was quantitatively assessed using fractional shortening calculations from M-mode measurements obtained from parasternal long-axis views and ejection fraction calculated using the modified Simpson’s method when adequate endocardial visualization was achieved. Systematic pulmonary artery evaluation included diameter measurements at standardized locations using two-dimensional imaging, with pulmonary annulus diameter measured in systole from parasternal short-axis view, main pulmonary artery diameter measured immediately distal to the pulmonary valve, and branch pulmonary artery assessment performed from suprasternal and high parasternal views when adequately visualized. Instances of incomplete visualization due to acoustic window limitations, patient factors, or complex post-surgical anatomy were systematically documented.

Hemodynamic assessment included measurement of pressure gradients using continuous-wave and pulsed-wave Doppler techniques, with neo-pulmonary valve gradients obtained using continuous-wave Doppler from multiple acoustic windows and peak instantaneous gradients calculated using the simplified Bernoulli equation. Complete aortic evaluation included systematic measurement of diameters at multiple levels using standardized two-dimensional imaging techniques, with aortic annulus diameter measured during systole from parasternal long-axis view, sinus of Valsalva diameter measured at maximal dimension, sinotubular junction diameter measured at the narrowest point, and ascending aorta diameter measured at its maximal dimension above the sinotubular junction. Hemodynamic assessment included measurement of peak pressure gradients across the neo-aortic valve using continuous-wave Doppler and assessment of aortic valve regurgitation using color Doppler mapping graded according to established criteria. Echocardiographic stenosis assessment was based on peak instantaneous pressure gradients calculated using the simplified Bernoulli equation (ΔP = 4v^2^). Pulmonary stenosis was graded as follows: trivial (<20 mmHg), mild (20–35 mmHg), moderate (36–60 mmHg), and severe (>60 mmHg). Neo-aortic stenosis was classified as trivial (<20 mmHg), mild (20–39 mmHg), moderate (40–64 mmHg), and severe (≥65 mmHg). Branch pulmonary artery stenosis was considered significant when peak systolic velocity exceeded 2.5 m/s or when there was aliasing on color Doppler examination with post-stenotic dilatation. Coronary artery anatomy was evaluated when possible using parasternal short-axis views with attention to visualization of both semilunar valves and coronary artery origins, though limitations of acoustic window accessibility were acknowledged and documented.

All measurements were performed by experienced observers using standardized protocols with Z-scores calculated using established normative values: Detroit normative data for pulmonary artery measurements, nomograms for aortic root diameters in children using two-dimensional echocardiography for aortic measurements, and Lopez et al. normative values for coronary artery dimensions, all adjusted for age and body surface area [27,28,29].

## 3. Statistical Analysis

Statistical analysis was performed using SPSS version 28.0 (IBM Corporation, Armonk, NY, USA). To minimize interobserver variability, all measurements were performed by experienced operators using standardized protocols. TTE measurements were conducted by pediatric cardiologists with more than 10 years of specialized experience, while CT measurements were performed by cardiac radiologists with extensive cardiac imaging experience, all following institutional standardized measurement guidelines. All continuous variables were first tested for normality using the Shapiro–Wilk test. Normally distributed variables were presented as mean ± standard deviation, while non-normally distributed data were presented as median with interquartile range (IQR). Categorical variables were expressed as frequencies and percentages with exact binomial 95% confidence intervals for prevalence estimates. Correlations between continuous variables were assessed using Spearman rank correlation coefficient due to the non-parametric nature of most measurements. Correlations of clinical interest were examined between PA bending angles and aortic root dimensions. Given the multiple correlations tested between PA bending angles and various aortic measurements, the Benjamini–Hochberg procedure was applied to these analyses to control the false discovery rate at 5% to account for multiple comparisons.

Comparisons between paired CT and echocardiographic measurements were performed using Wilcoxon signed-rank test for non-normally distributed data and paired *t*-test for normally distributed variables. Agreement between imaging modalities was evaluated using Bland–Altman analysis with calculation of mean bias and 95% limits of agreement. The proportion of measurements falling within the limits of agreement was calculated to assess clinical acceptability. Diagnostic completeness was compared between modalities using McNemar’s test for paired proportions. The diagnostic yield of each imaging modality was calculated as the percentage of patients in whom complete anatomical assessment was achieved. For radiation dose analysis, effective dose calculations were performed using age-specific conversion factors, and dose-length product values were analyzed using descriptive statistics. Correlations between patient anthropometric variables and radiation exposure were assessed to identify dose optimization opportunities.

A two-tailed *p*-value less than 0.05 was considered statistically significant for all analyses. All statistical tests were performed with appropriate assumptions verification, and non-parametric alternatives were used when distributional assumptions were violated.

## 4. Results

Forty-seven d-TGA patients post-ASO were included. Median follow-up age was 37.2 months (IQR: 8.4–128.8) with 66% male predominance. ASO was performed at median 9 days (IQR: 3–14). Growth parameters were normal. Median radiation exposure was 2.684 mSv (IQR: 1.5–4.6). Patient characteristics are in Table 1.

**Figure 1:**
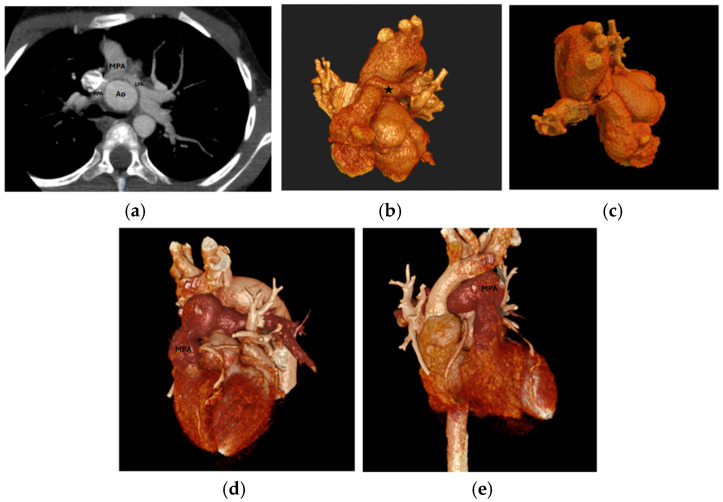
(**a**) LPA and RPA stenosis on axial plane, (**b**) 3D RPA stenosis, Star: the location of 3D RPA stenosis. (**c**) 3D LPA stenosis, (**d**,**e**) 3D MPA stenosis. LPA: Left Pulmonary Artery, RPA: Right Pulmonary Artery, MPA: Main Pulmonary Artery, 3D: Three-dimensional; Ao: Aorta.

**Figure 2:**
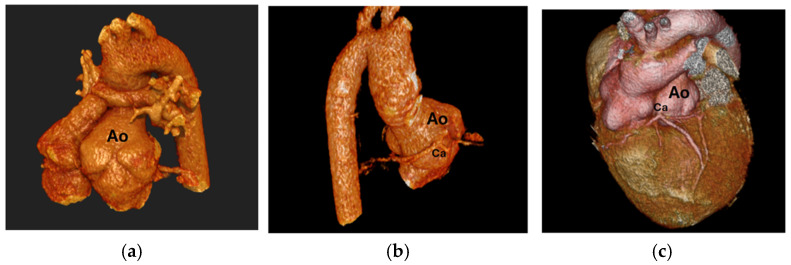
(**a**) Dilated aortic root, (**b**) Coronary artery coursing posterior to the aorta, (**c**) Single root coronary artery anomaly, Ao: Aorta, Ca: Coronary artery.

**Figure 3:**
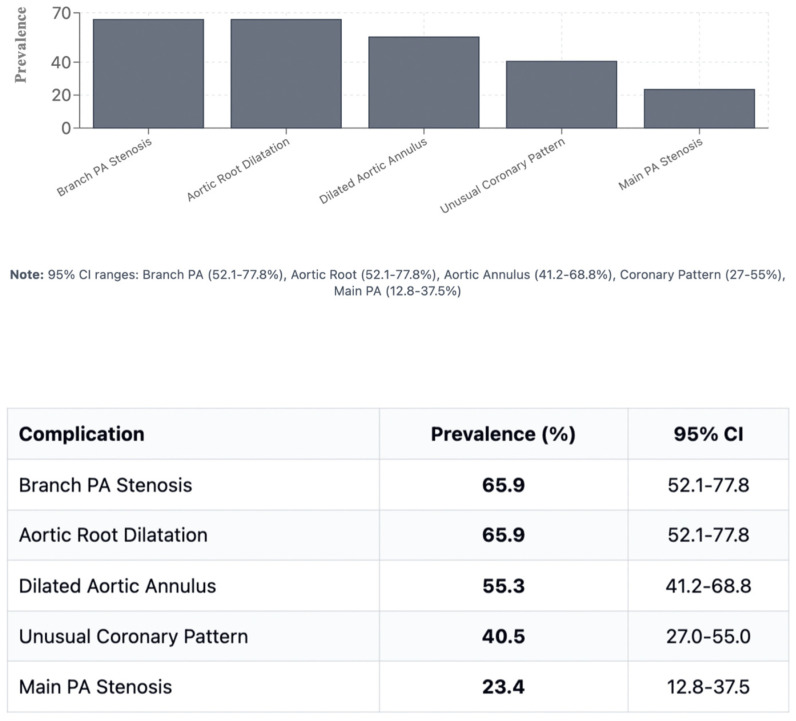
Prevalence of major post-ASO complications. PA: pulmonary artery; SOV: sinus of Valsalva; STJ: sinotubular junction; MAPCA: major aortopulmonary collateral artery.

Branch PA stenosis was detected by CT in 31 patients (65.9%), representing the most common post-ASO complication, with bilateral stenosis in 12 cases (25.5%) and unilateral stenosis in 19 cases (40.4%). Quantitative measurements revealed significant PA undersizing, with median Z-scores substantially below normal: main PA −1.62, RPA −1.29, and LPA −1.79. Novel geometric measurements showed abnormal PA bending patterns (RPA: 98.45° ± 19.82°, LPA: 92.45° ± 16.89°). Main pulmonary artery, right and left pulmonary artery stenosis are shown in Figure 1. Aortic root dilatation was prevalent, with dilated aortic annulus in 26 patients (55.3%), dilated aortic root in 31 patients (65.9%), and dilated sinotubular junction in 22 patients (46.8%). Median Z-scores significantly exceeded normal values: aortic annulus 2.95, sinus of Valsalva 2.56, and sinotubular junction 2.47. Normal coronary anatomy was present in 28 patients (59.5%), with unusual coronary patterns in 19 patients (40.5%). MAPCAs were detected in 6 patients (12.7%). Dilated aortic root and coronary abnormalities are shown in Figure 2. CT angiography findings are presented in Table 2. The prevalence of major post-ASO complications is illustrated in Figure 3.

Echocardiography showed preserved left ventricular function (median fractional shortening 34.1%, IQR: 32.2–39.7%). Neo-aortic regurgitation occurred in 27 patients (57.4%) and neo-pulmonary stenosis in 40 patients (85.1%), mostly mild. However, TTE provided incomplete assessment in 11 patients (23.4%) for pulmonary arteries and 13 patients (27.6%) for coronary evaluation. Echocardiographic findings are summarized in Table 3.

**Figure 4:**
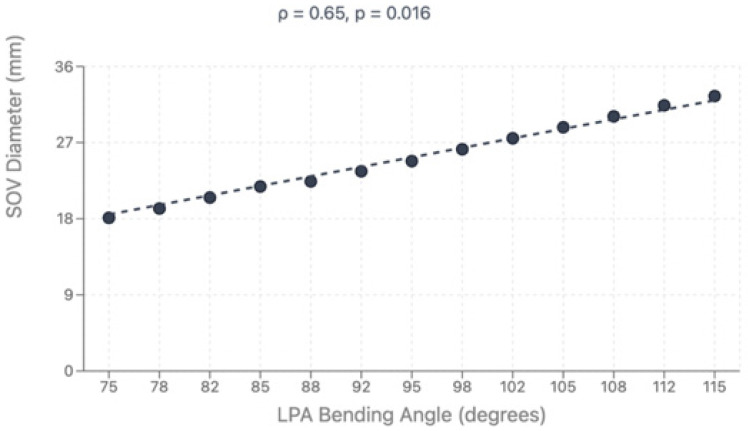
Bland–Altman plot showing agreement between CTA and TTE. CTA: computed tomography, TTE: transthoracic echocardiography.

**Figure 5:**
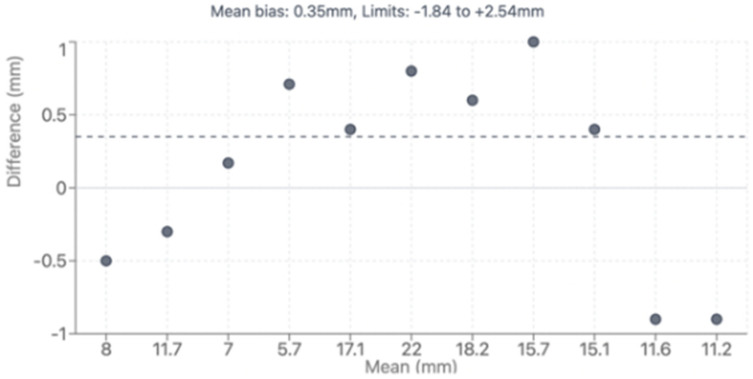
Correlation between LPA bending angle and sinus of Valsalva diameter.

Direct comparison between modalities revealed good correlation for major vessel dimensions when both provided adequate visualization (*p* > 0.05 for all major comparisons). Complete CT analysis was achieved in all 47 patients (100%), while echocardiographic assessment provided complete pulmonary artery evaluation in 36 patients (76.6%) and complete coronary evaluation in 34 patients (72.4%). Bland–Altman analysis demonstrated acceptable agreement with mean bias of 0.35mm and 95% limits of agreement from −1.84 to +2.54 mm, as shown in Figure 4. However, CT demonstrated superior diagnostic completeness, successfully evaluating all patients’ anatomy (100%) compared to echocardiography’s incomplete assessment in 23.4% of PA cases and 27.6% of coronary cases. Direct comparison between modalities is presented in Table 4. LPA bending angle showed strong positive correlation with sinus of Valsalva diameter (rho = 0.65, *p* = 0.016) and Z-score (rho = 0.69, *p* = 0.021), suggesting that progressive aortic root dilatation influences pulmonary artery geometry, particularly affecting LPA trajectory as it courses around the enlarged neo-aortic root. Analysis of geometric correlations is detailed in Table 5 and illustrated in Figure 4 and Figure 5.

**Figure 6:**
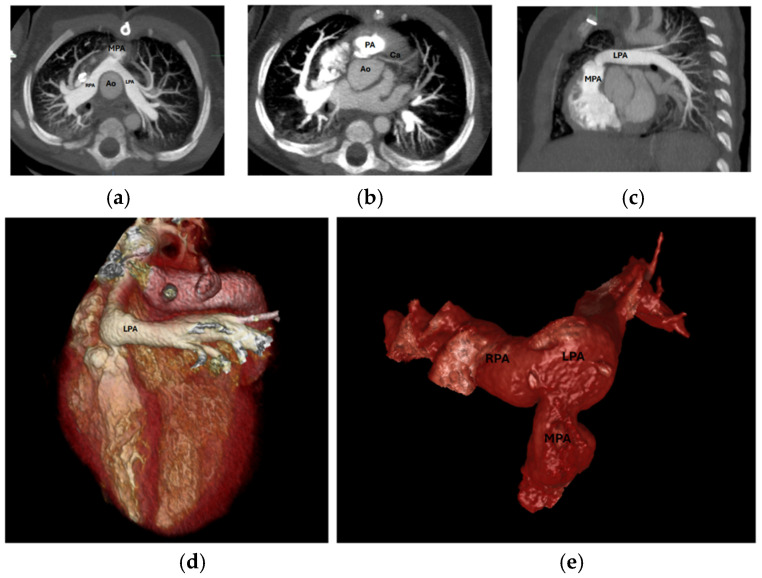
(**a**) Axial MPA stenosis, (**b**) Axial coronary artery origin, (**c**): Sagittal LPA stenosis, (**d**) 3D LPA stenosis, (**e**) 3D PA and branches. MPA: Main Pulmonary Artery, LPA: Left Pulmonary Artery, PA: Pulmonary Artery, 3D: Three-dimensional, Ao: Aorta, RPA: Right Pulmonary Artery, Ca: Coronary artery.

During follow-up, 10 patients (21.3%) required cardiovascular interventions: 3 patients underwent neo-aortic valve interventions (2 repairs, 1 replacement), and 7 patients required PA interventions (6 percutaneous balloon angioplasty, 1 surgical). Among PA intervention cases, 7 patients (100%) had incomplete echocardiographic PA assessment, and intervention decisions were guided by combined TTE and CT findings. One patient required surgical rather than percutaneous approach due to high-risk coronary–pulmonary proximity identified by CTMain pulmonary artery, left pulmonary artery stenosis and coronary origin between the main pulmonary artery and aorta are shown in Figure 6. Clinical interventions and imaging assessment are detailed in Table 6.

## 5. Discussion

This study demonstrates that while transthoracic echocardiography (TTE) remains valuable for functional assessment and routine surveillance following arterial switch operation (ASO), it has significant limitations in providing complete anatomical evaluation. Cardiac CT angiography emerges as an essential complementary imaging modality, offering superior anatomical detail particularly for pulmonary arteries, coronary anatomy, and complex spatial relationships critical for long-term management.

Our findings reveal a high prevalence of PA abnormalities (65.9%), confirming PA stenosis as the most common long-term complication after ASO. This prevalence aligns with recent large cohort studies reporting PA complications in 50–80% of patients following ASO [12,30,31]. The prevalence of PA stenosis in our cohort (65.9%) aligns remarkably with the recent findings of Rakha et al., who reported identical rates of 65% in their smaller cohort [32]. This consistency across different patient populations and institutions strengthens the evidence for PA stenosis as the predominant long-term complication following ASO. A recent multicenter study of 7411 infants demonstrated that 17% required PA intervention with a median time to intervention of 0.8 years [12]. The predominance of RPA stenosis over LPA stenosis (23.4% vs. 17.0%) in our series differs from some reports. Morgan et al. found LPA stenosis more common, attributed to compression by the enlarged aorta [17]. These variations likely reflect differences in surgical technique, patient demographics, follow-up duration, and the complex interaction between neo-aortic root geometry and PA positioning after the Lecompte maneuver.

The inability of echocardiography to adequately assess PAs in 23.4% of patients represents a critical diagnostic limitation. Rakha et al. reported incomplete PA visualization in 35% of cases and incomplete coronary assessment in 40% of patients, closely paralleling our observed rates of 23.4% and 27.6%, respectively [32]. This consistency across multiple centers confirms the universal nature of these diagnostic limitations. This is particularly concerning given that PA stenosis often requires reintervention and significantly impacts long-term functional outcomes, exercise capacity, and quality of life [33,34]. Recent studies have highlighted the complex relationship between PA stenosis and inefficient flow patterns rather than stenosis alone. Delaney et al. demonstrated that right ventricular afterload in repaired d-TGA is associated with inefficient flow patterns, emphasizing the importance of detailed anatomical assessment [35]. The novel geometric measurements in our study, including PA bending angles, provide quantitative insight into three-dimensional alterations created by the Lecompte maneuver.

Aortic root dilatation was observed in 65.9% of our cohort, with median Z-scores significantly exceeding normal values across all measured segments. This prevalence is consistent with recent longitudinal studies. Van der Palen et al. demonstrated that neo-aortic dimensions continue to increase in adulthood without stabilization, with annual diameter increases of 0.39 ± 0.06 mm for neo-aortic annulus, 0.63 ± 0.09 mm for root, and 0.54 ± 0.11 mm for sinotubular junction [16]. A comprehensive meta-analysis by Jacquemyn et al. of 6169 TGA patients after ASO found that neo-aortic root dilatation is present in half of children and the majority of adults (34.9% freedom from neo-aortic root dilatation at 20 years) [36]. The pathophysiology involves multiple factors including intrinsic differences in pulmonary root histology compared to native aortic tissue, surgical trauma disrupting vasa vasorum, altered hemodynamics with abnormal flow patterns, and geometric distortion from coronary translocation [37,38]. Recent computational fluid dynamics studies have demonstrated increased wall shear stress in post-ASO aortic geometries compared to normal controls, contributing to progressive dilatation [39]. Our finding of geometric correlations between aortic root dimensions and PA bending angles provides new insight into the interconnected nature of post-ASO anatomical changes. The significant correlation between LPA bending angle and aortic root dimensions (rho = 0.65, *p* = 0.016) suggests that progressive aortic root dilatation creates secondary mechanical effects on adjacent structures, potentially contributing to progressive PA distortion over time.

Coronary artery evaluation represents one of the most compelling diagnostic advantages of CT over echocardiography in post-ASO surveillance. While echocardiography failed to provide complete coronary assessment in 27.6% of patients, CT successfully characterized coronary anatomy in all cases. This capability is crucial given the critical importance of coronary complications in determining long-term outcomes. Recent systematic reviews of adults after ASO found that while the overall incidence of coronary events is low (0.4% intervention rate, 0.4% coronary death), anatomical high-risk features are common, including stenosis (4%), acute angles (40%), kinking (24%), and inter-arterial course (11%) [11]. Batteux et al. used 3D modeling to define geometric criteria associated with coronary events, finding that four out of six criteria of left coronary artery geometry were associated with coronary events: clockwise position > 67°, first centimeter angle > 62°, minimal 3D angle < 39°, and distance between coronary ostium and PA < 1 mm [40]. These sophisticated analyses underscore the importance of detailed anatomical assessment possible only with high-resolution CT imaging. The prevalence of unusual coronary patterns in 40.5% of our patients reflects both the inherent anatomical complexity of TGA coronary distributions and potential surgical alterations. Current guidelines from both ACC/AHA and ESC recommend at least baseline coronary assessment, though the optimal surveillance strategy remains debated [19,22].

The median radiation exposure of 2.684 mSv in our cohort demonstrates successful implementation of contemporary dose optimization strategies. This exposure level is comparable to approximately 11 months of natural background radiation and represents a favorable risk-benefit ratio considering the comprehensive diagnostic information obtained. Modern CT techniques, including prospective ECG gating, automatic tube current modulation, iterative reconstruction algorithms, and weight-based kV selection, have dramatically reduced radiation doses while maintaining image quality [41,42]. The implementation of weight-based protocols (80 kV for patients < 35 kg, 100 kV for patients > 35 kg) optimizes iodine contrast enhancement while minimizing radiation exposure. Additionally, the comprehensive information obtained from a single CT study may reduce the need for multiple follow-up echocardiograms and cardiac catheterizations, potentially offsetting the higher initial cost and providing long-term economic benefits. Despite CT’s diagnostic advantages, several practical limitations must be acknowledged. Cost considerations include higher examination fees compared to echocardiography and potential need for sedation in younger children, increasing overall healthcare costs. Accessibility remains limited in some healthcare settings, particularly in resource-limited environments where advanced CT technology and specialized pediatric cardiac imaging expertise may not be readily available. Additionally, concerns regarding cumulative radiation exposure over lifelong surveillance protocols require careful consideration, though modern dose optimization techniques substantially reduce this risk.

Rather than replacing echocardiography, our findings support CT as a complementary modality addressing specific diagnostic gaps in post-ASO surveillance. This approach aligns with recent multimodality imaging guidelines emphasizing the complementary nature of different imaging techniques [18]. Echocardiography excels in functional assessment, providing real-time hemodynamic information, detailed valve function evaluation, ventricular performance assessment, and dynamic flow evaluation. Its widespread availability, portability, lack of radiation exposure, and ability to provide immediate results make it ideal for routine surveillance and acute clinical scenarios. While cardiac MRI offers radiation-free imaging with excellent soft tissue contrast, CT maintains specific advantages in post-ASO surveillance including superior spatial resolution for coronary anatomy, faster acquisition reducing sedation needs, and better visualization of calcifications. The modality choice should consider patient age, sedation requirements, radiation concerns, and specific anatomical questions being addressed. CT’s primary strengths lie in comprehensive anatomical assessment, particularly for structures poorly visualized by echocardiography due to acoustic limitations, complex geometry, or post-surgical anatomy. Recent studies have confirmed CT’s superior ability to assess PA anatomy, coronary origins and courses, and complex three-dimensional relationships in post-ASO patients [11,43].

The intervention rate (21.3%) in our cohort demonstrates the clinical significance of accurate anatomical assessment in post-ASO management and aligns with recent large cohort studies reporting reintervention rates of 13–20% [8,10]. CT demonstrated superior utility in intervention planning, providing complete anatomical evaluation in all cases compared to incomplete TTE assessment in 20% of intervention candidates. Among PA interventions, 2 patients (28.6%) had incomplete echocardiographic assessment, while CT successfully guided intervention planning in all cases, consistent with published reports emphasizing CT’s superior anatomical visualization capabilities in post-ASO surveillance [20,21]. The identification of high-risk coronary–pulmonary proximity by CT in one patient prevented potential procedural complications and guided appropriate surgical management, aligning with recent studies highlighting the increased procedural risks in post-ASO PA interventions compared to other congenital conditions [12,44]. These findings support CT’s integration into surveillance protocols, particularly when intervention is being considered or TTE assessment is incomplete, directly impacting patient safety and procedural planning as recommended by current multimodality imaging guidelines [45].

The comprehensive anatomical information provided by CT has direct implications for clinical decision-making across multiple domains. Accurate PA assessment guides decisions regarding need for, timing of, and approach to catheter-based or surgical interventions [46]. Recent data suggests that percutaneous PA interventions in post-ASO patients carry higher risks than in other conditions, making accurate anatomical assessment critical [30]. Detailed coronary artery evaluation informs risk stratification for activity recommendations, competitive sports participation, and need for additional cardiac testing. While sudden cardiac death due to coronary complications is extremely rare (no proven cases in 8798 patients with 66,450 patient follow-up years in a recent systematic review), the ability to identify high-risk anatomical features remains important for optimal care [44].

Based on current evidence and our findings, we propose an integrated imaging approach where echocardiography serves as the primary screening and functional assessment tool, with CT reserved for complete anatomical evaluation at appropriate intervals or when echocardiographic assessment is incomplete. This strategy optimizes diagnostic accuracy while minimizing radiation exposure, healthcare costs, and patient burden. We suggest CT evaluation at key developmental stages: school age (6–8 years), adolescence (14–16 years), and transition to adult care (18–20 years), with additional studies based on echocardiographic findings, clinical symptoms, or concerning anatomical features. Given the progressive nature of post-ASO complications, periodic detailed anatomical assessment appears warranted, particularly as patients transition to adult care where the cumulative burden of late complications becomes more significant.

Future research should focus on establishing evidence-based protocols for CT surveillance intervals, correlating anatomical findings with functional outcomes, and developing risk stratification algorithms incorporating both anatomical and functional parameters. Large multicenter prospective studies are needed to validate our proposed imaging protocol and establish long-term clinical outcomes.

## 6. Limitations

This study has several limitations. The single-center retrospective design limits generalizability, and the modest sample size (*n* = 47) may affect statistical power for some analyses. The absence of long-term clinical outcome data limits assessment of the prognostic significance of anatomical abnormalities detected by either modality. Additionally, systematic inter-observer variability analysis was not performed for all echocardiographic measurements, which may affect the reliability of comparative assessments. Finally, cost-effectiveness analysis of the proposed integrated imaging approach was not conducted, which would be valuable for clinical implementation.

## 7. Conclusions

This study demonstrates that transthoracic echocardiography alone is insufficient for complete anatomical assessment following arterial switch operation in patients with d-TGA. While TTE remains essential for functional evaluation, it provided incomplete pulmonary artery assessment in 23.4% and incomplete coronary evaluation in 27.6% of patients. Cardiac CT angiography offers superior anatomical visualization with complete diagnostic capability in all patients. The high prevalence of complications detected by CT—including pulmonary artery stenosis (65.9%), aortic root dilatation (65.9%), and coronary anomalies (40.5%)—underscores the clinical importance of comprehensive imaging. CT’s critical role is evidenced by successful guidance of all cardiovascular interventions (21.3% of patients) and identification of high-risk anatomy that altered surgical approach. An integrated imaging approach utilizing TTE for functional assessment and CT for detailed anatomical evaluation at appropriate intervals optimizes post-ASO surveillance. CT should be considered at key developmental stages and when TTE assessment is incomplete or intervention is being considered. Cardiac CT angiography should be considered an essential component of comprehensive post-ASO surveillance protocols, particularly when detailed anatomical information is required for clinical decision-making.

## Figures and Tables

**Table 1:** Patient Demographics and Clinical Characteristics.

Parameter	Value
**Demographics**	
Sex (male), *n* (%)	31 (66%)
Age at follow-up (months)	37.2 (8.4–128.8)
Weight (kg)	14.7 (7.4–36.2)
Weight (percentile)	32.1 (22.3–47.1)
Weight (SDS)	−0.29 (−1.07–1.16)
Height (cm)	97.8 (73.6–128.9)
Height (percentile)	35.19 (24.9–51.6)
Height (SDS)	0.11 (−0.89–1.24)
BMI (kg/m^2^)	17.6 (12.1–19.4)
BMI (percentile)	28.75 (23.9–41.12)
BMI (SDS)	−0.53 (−1.26–0.87)
Surgical Variables	
ASO Age (days)	9 (3–14)
Pre-operative septostomy, *n* (%)	12 (25.5%)
Imaging Parameters	
Contrast agent (cc/kg)	4.5 (3.2–6.9)
Radiation dose (mSv)	2.684 (1.5–4.6)

Data presented as median (IQR) or *n* (%). SDS: Standard Deviation Score, BMI: Body Mass Index, ASO: Arterial Switch Operation.

**Table 2:** Cardiac CT Angiography Findings.

Parameter	Value
**Pulmonary Arteries**	
Pulmonary Annulus (mm)	14.7 (8.1–16.7)
Pulmonary Annulus Z Score	0.26 (−1.12–0.79)
Main PA Diameter (mm)	11.5 (7.9–15.9)
Main PA Diameter Z Score	−1.62 (−0.68 to −2.12)
RPA Diameter (mm)	7.12 (4.91–9.8)
RPA Diameter Z score	−1.29 (−0.79 to −2.27)
LPA Diameter (mm)	6.08 (4.32–8.5)
LPA Diameter Z score	−1.79 (−0.62 to −2.51)
RPA area (mm^2^/m^2^)	91.87 (49.56–120.94)
LPA area (mm^2^/m^2^)	74.19 (42.71–101.35)
Main PA axial Angle (degree)	2.1 (−12.1–6.6)
RPA bending Angle (degree)	98.45 ± 19.82
LPA bending Angle (degree)	92.45 ± 16.89
**Pulmonary Stenosis**	
Branch PA stenosis, *n* (%)	31 (65.9%)
Bilateral PAs stenosis, *n* (%)	12 (25.5%)
Unilateral PA stenosis, *n* (%)	19 (40.4%)
Unilateral RPA stenosis, *n* (%)	11 (23.4%)
Unilateral LPA stenosis, *n* (%)	8 (17.0%)
Main PA stenosis, *n* (%)	11 (23.4%)
**Aortic Measurements**	
Aortic annulus Diameter (mm)	17.3 (13.5–23.9)
Aortic annulus Z score	2.95 (1.78–3.86)
SOV Diameter (mm)	22.4 (18.1–32.5)
SOV Z score	2.56 (1.56–3.11)
SOV area (mm^2^/m^2^)	594.25 (467.25–723.53)
STJ Diameter (mm)	18.5 (15.1–25.8)
STJ Z score	2.47 (1.65–3.04)
STJ area (mm^2^/m^2^)	496.26 (412.29–547.89)
Ascending aorta Diameter (mm)	16.2 (14.9–24.1)
Ascending aorta Z score	1.03 (−0.12–1.65)
Ascending aorta area (mm^2^/m^2^)	275.49 (241.12–329.86)
Transverse Arch (mm)	15.3 (11–21.29)
Transverse Arch Z score	1.09 (−0.67–1.92)
Distal Arch (mm)	11.2 (8.1–15.2)
Distal Arch Z score	0.76 (0.12–1.71)
Isthmus Diameter (mm)	10.8 (8.3–15.4)
Isthmus Z score	0.75 (0.19–1.58)
**Aortic Abnormalities**	
Dilated Aortic annulus, *n* (%)	26 (55.3%)
Dilated Aortic Root, *n* (%)	31 (65.9%)
Dilated STJ, *n* (%)	22 (46.8%)
Dilated Ascending aorta, *n* (%)	19 (40.4%)
Supravalvular aortic stenosis, *n* (%)	3 (6.5%)
Aortic coarctation/arch abnormalities, *n* (%)	2 (4.3%)
APCAs or MAPCAs, *n* (%)	6 (12.7%)
**Coronary Patterns**	
Normal coronary artery (1LCx-2R), *n* (%)	28 (59.5%)
Unusual coronary patterns, *n* (%)	19 (40.5%)
- 1L-2RCx, *n* (%)	8 (17.0%)
- 2LCxR, *n* (%)	4 (8.5%)
- 1R-2LCx, *n* (%)	3 (6.4%)
- 1LR-2Cx, *n* (%)	2 (4.3%)
- 1RCxL, *n* (%)	1 (2.1%)

Data presented as median (IQR), mean ± SD, or *n* (%). SOV: Sinus of Valsalva, STJ: Sinotubular junction, RPA: Right Pulmonary Artery, LPA: Left Pulmonary Artery, APCA: Aortopulmonary Collateral Artery, MAPCA: Major Aortopulmonary Collateral Artery.

**Table 3:** Transthoracic Echocardiography Findings.

Parameter	Value
**Pulmonary Assessment**	
Neo-pulmonary valve pressure gradient (mmHg)	14.7 (11.4–31.6)
**Neo-pulmonary stenosis, *n* (%)**	
- Trivial	14 (29.7%)
- Mild	16 (34.0%)
- Moderate	4 (8.5%)
- Severe	6 (12.8%)
**Neo-pulmonary regurgitation, *n* (%)**	
- Trivial	16 (34.0%)
- Mild	7 (14.9%)
- Moderate	2 (4.3%)
- Severe	1 (2.1%)
RPA pressure gradient (mmHg)	15.2 (12.9–26.8)
LPA pressure gradient (mmHg)	18.9 (14.2–31.5)
Pulmonary Annulus (mm)	15.1 (7.9–17.1)
Pulmonary Annulus Z Score	0.34 (−1.01–0.65)
Main PA Diameter (mm)	11.8 (7.7–15.3)
Main PA Diameter Z score	−1.45 (−0.78 to −2.27)
RPA Diameter (mm)	6.95 (4.57–10.23)
RPA Diameter Z score	−1.09 (−0.68 to −2.37)
LPA Diameter (mm)	5.37 (4.29–8.96)
LPA Diameter Z score	−1.46 (−0.57 to −2.64)
Pulmonary not evaluated, *n* (%)	11 (23.4%)
**Aortic Assessment**	
Neo-aortic valve pressure gradient (mmHg)	9.2 (4.5–14.3)
**Neo-aortic Regurgitation, *n* (%)**	
- Trivial	16 (34.0%)
- Mild	4 (8.5%)
- Moderate	4 (8.5%)
- Severe	3 (6.4%)
**Neo-aortic stenosis, *n* (%)**	
- Trivial	9 (19.1%)
- Mild	4 (8.5%)
- Moderate	2 (4.3%)
- Severe	3 (6.4%)
Aortic coarctation/abnormalities, *n* (%)	5 (10.6%)
Aortic annulus Diameter (mm)	16.9 (12.1–25.6)
Aortic annulus Z score	2.62 (1.65–3.65)
SOV Diameter (mm)	21.6 (16.9–34.5)
SOV Z score	2.32 (1.45–3.21)
STJ Diameter (mm)	17.9 (14.1–26.8)
STJ Z score	2.35 (1.52–3.24)
Ascending aorta Diameter (mm)	15.2 (13.9–23.1)
Ascending aorta Z score	0.89 (−0.24–1.55)
Transverse Arch (mm)	14.9 (11.3–20.12)
Transverse Arch Z score	0.98 (−0.57–1.72)
Distal Arch (mm)	12.1 (7.9–16.1)
Distal Arch Z score	0.94 (0.32–1.96)
Isthmus Diameter (mm)	11.7 (7.8–16.9)
Isthmus Z score	0.82 (0.21–1.63)
**Aortic Abnormalities**	
Dilated Aortic annulus, *n* (%)	21 (44.6%)
Dilated Aortic Root, *n* (%)	24 (51.0%)
Dilated STJ, *n* (%)	19 (40.4%)
Dilated Ascending aorta, *n* (%)	17 (36.2%)
Supravalvular aortic stenosis, *n* (%)	4 (8.5%)
Aortic coarctation/arch abnormalities, *n* (%)	3 (6.4%)
APCAs or MAPCAs, *n* (%)	8 (17.0%)
**Coronary Assessment**	
Normal coronary artery, *n* (%)	14 (29.7%)
Unusual coronary artery, *n* (%)	20 (42.5%)
Not evaluated, *n* (%)	13 (27.6%)
**Ventricular Function**	
LV FS (%)	34.12 (32.19–39.65)

Data presented as median (IQR) or *n* (%). SOV: Sinus of Valsalva, STJ: Sinotubular junction, LV FS: Left Ventricular Fractional Shortening, RPA: Right Pulmonary Artery, LPA: Left Pulmonary Artery, APCA: Aortopulmonary Collateral Artery, MAPCA: Major Aortopulmonary Collateral Artery.

**Table 4:** Comparison of CT and Echocardiography Measurements.

Parameter	CT	Echocardiography	*p* Value
**Pulmonary Measurements**			
Pulmonary Annulus (mm)	14.7 (8.1–16.7)	15.1 (7.9–17.1)	0.44
Pulmonary Annulus Z Score	0.26 (−1.12–0.79)	0.34 (−1.01–0.65)	0.53
Main PA Diameter (mm)	11.5 (7.9–15.9)	11.8 (7.7–15.3)	0.31
Main PA Diameter Z Score	−1.62 (−0.68 to −2.12)	−1.45 (−0.78 to −2.27)	0.67
RPA Diameter (mm)	7.12 (4.91–9.8)	6.95 (4.57–10.23)	0.76
RPA Diameter Z score	−1.29 (−0.79 to −2.27)	−1.09 (−0.68 to −2.37)	0.54
LPA Diameter (mm)	6.08 (4.32–8.5)	5.37 (4.29–8.96)	0.27
LPA Diameter Z score	−1.79 (−0.62 to −2.51)	−1.46 (−0.57 to −2.64)	0.46
**Aortic Measurements**			
Aortic annulus Diameter (mm)	17.3 (13.5–23.9)	16.9 (12.1–25.6)	0.39
Aortic annulus Z score	2.95 (1.78–3.86)	2.62 (1.65–3.65)	0.54
SOV Diameter (mm)	22.4 (18.1–32.5)	21.6 (16.9–34.5)	0.47
SOV Z score	2.56 (1.56–3.11)	2.32 (1.45–3.21)	0.13
STJ Diameter (mm)	18.5 (15.1–25.8)	17.9 (14.1–26.8)	0.32
STJ Z score	2.47 (1.65–3.04)	2.35 (1.52–3.24)	0.49
Ascending aorta Diameter (mm)	16.2 (14.9–24.1)	15.2 (13.9–23.1)	0.17
Ascending aorta Z score	1.03 (−0.12–1.65)	0.89 (−0.24–1.55)	0.29
Transverse Arch (mm)	15.3 (11–21.29)	14.9 (11.3–20.12)	0.52
Transverse Arch Z score	1.09 (−0.67–1.92)	0.98 (−0.57–1.72)	0.73
Distal Arch (mm)	11.2 (8.1–15.2)	12.1 (7.9–16.1)	0.61
Distal Arch Z score	0.76 (0.12–1.71)	0.94 (0.32–1.96)	0.59
Isthmus Diameter (mm)	10.8 (8.3–15.4)	11.7 (7.8–16.9)	0.37
Isthmus Z score	0.75 (0.19–1.58)	0.82 (0.21–1.63)	0.53

Data presented as median (IQR). SOV: Sinus of Valsalva, STJ: Sinotubular junction, RPA: Right Pulmonary Artery, LPA: Left Pulmonary Artery.

**Table 5:** Correlations Between PA Bending Angles and Aortic Measurements.

Parameter	RPA Bending Angle	*p*	LPA Bending Angle	*p*
	Rho		Rho	
Aortic annulus (mm)	−0.37	0.08	0.21	0.49
Aortic annulus Z score	−0.28	0.56	0.11	0.72
SOV (mm)	−0.19	0.33	0.65	**0.016**
SOV Z score	0.05	0.47	0.69	**0.021**
STJ (mm)	0.13	0.67	0.53	**0.038**
STJ Z score	0.16	0.31	0.42	**0.042**
Ascending Aorta (mm)	−0.11	0.32	0.36	0.23
Ascending Aorta Z score	0.24	0.34	0.53	0.06
Transverse Arch (mm)	0.19	0.21	0.42	0.05
Transverse Arch Z score	0.06	0.38	0.38	0.42
Isthmus (mm)	0.09	0.65	0.54	0.11
Isthmus Z score	0.31	0.43	0.64	0.19

SOV: Sinus of Valsalva, STJ: Sinotubular junction, RPA: Right Pulmonary Artery, LPA: Left Pulmonary Artery. Bold values indicate statistical significance after Benjamini–Hochberg correction for multiple comparisons (q < 0.05).

**Table 6:** Clinical Interventions and Imaging Assessment.

Parameter	CT Assessment	TTE Assessment	Total
**Aortic Valve Interventions**			
- Complete pre-intervention assessment	3 (100%)	3 (100%)	3
- Valve repair	2	2	2
- Valve replacement	1	1	1
**Pulmonary Artery Interventions**			
- Complete pre-intervention assessment	7 (100%)	0 (0%)	7
- Percutaneous balloon angioplasty	6	-	6
- Surgical intervention	1	-	1
**Intervention sites**			
- RPA + LPA + MPA	2	0 (0%)	2
- LPA only	2	0 (0%)	2
- RPA only	1	0 (0%)	1
- LPA + RPA	1	0 (0%)	1
- Surgical PA intervention	1	0 (0%)	1
**High-risk anatomy identification**			
- Coronary-PA proximity (surgical indication)	1 (100%)	0 (0%)	1
**Total patients requiring intervention**	10 (21.3%)	3 (30%)	10

## Data Availability

The data that support the findings of this study are available from the corresponding author upon reasonable request.
